# Electrocardiographic Left Ventricular Mass Trajectories and the Effects of Treatment in Patients at Different Stages of Hypertension

**DOI:** 10.3390/jcm14072313

**Published:** 2025-03-28

**Authors:** Flávio D. Fuchs, Leonardo Krause Valter, Arthur Lacerda Tavares, Beatriz Padoin Camilo, Paul K. Whelton, Luiz C. N. Scala, José F. Vilela-Martin, Carlos E. Poli-de-Figueiredo, Ricardo Pereira e Silva, Miguel Gus, Luiz A. Bortolotto, Rosane P. Schlatter, Evandro J. Cesarino, Iran Castro, José A. Figueiredo Neto, André A. Steffens, João G. Alves, Andréa A. Brandão, Marcos R. de Sousa, Paulo C. Jardim, Leila B. Moreira, Roberto S. Franco, Marco M. Gomes, Felipe C. Fuchs, Dario Sobral Filho, Antônio C. Nóbrega, Fernando Nobre, Otávio Berwanger, Sandra C. Fuchs

**Affiliations:** 1 Division of Cardiology, Hospital de Clínicas de Porto Alegre, Universidade Federal do Rio Grande Do Sul, Ramiro Barcelos, 2350, Porto Alegre 90035-903, RS, Brazil; lvalter@hcpa.edu.br (L.K.V.); altavares@hcpa.edu.br (A.L.T.); bcamilo@hcpa.edu.br (B.P.C.); mgus@terra.com.br (M.G.); rschlatter@hcpa.edu.br (R.P.S.); lbmoreira@hcpa.edu.br (L.B.M.); felipefuchs@hcpa.edu.br (F.C.F.); sfuchs@hcpa.edu.br (S.C.F.); 2 Department of Epidemiology, School of Public Health and Tropical Medicine, Tulane University, New Orleans, LA 70112, USA; pkwhelton@gmail.com; 3 Hospital Universitário Júlio Müller, Universidade Federal de Mato Grosso, Cuiabá 78048-902, MT, Brazil; lscala@terra.com.br; 4 Faculdade de Medicina São José Do Rio Preto, São José do Rio Preto 15090-000, SP, Brazil; vilelamartin@uol.com.br; 5 Faculdade de Medicina Hospital São Lucas, PUCRS, Porto Alegre 90619-900, RS, Brazil; cepolif@pucrs.br; 6 Hospital Universitário Walter Cantídio, Universidade Federal do Ceará, Fortaleza 60430-372, CE, Brazil; ricardopereirasilva.ufc@gmail.com; 7 Faculdade de Medicina, Instituto do Coração, Universidade de São Paulo, São Paulo 05403-000, SP, Brazil; bortolotto1961@gmail.com; 8 Faculdade de Ciências Farmacêuticas, USP Ribeirão Preto, Ribeirão Prêto 14040-900, SP, Brazil; cesarino@fcfrp.usp.br; 9 Instituto de Cardiologia, Av. Princesa Isabel, Porto Alegre 90620-001, RS, Brazil; irancastro@gmail.com; 10 Hospital Universitário, Universidade Federal do Maranhão, São Luís 65085-580, MA, Brazil; jafneto@terra.com.br; 11 Hospital Universitário, Universidade Federal de Pelotas, Pelotas 96010-610, RS, Brazil; steffens.sul@terra.com.br; 12 Instituto de Medicina Integral Professor Fernando Figueira, Recife 50070-550, PE, Brazil; joaoguilherme@imip.org.br; 13 Universidade Do Estado do Rio de Janeiro, Rio de Janeiro 20551-030, RJ, Brazil; andreaaraujobrandao@gmail.com; 14 Hospital das Clínicas, Universidade Federal de Minas Gerais, Belo Horizonte 31270-901, MG, Brazil; sousa.mr@uol.com.br; 15 Hospital das Clínicas de Goiânia, Universidade Federal de Goiás, Goiânia 74605-050, GO, Brazil; fvjardim.ufg@gmail.com; 16 Faculdade de Medicina de Botucatu, Botucatu 18607-741, SP, Brazil; roberto.js.franco@unesp.br; 17 Hospital do Coração de Alagoas, Maceió 57052-580, AL, Brazil; mota-gomes@uol.com.br; 18 Hospital Universitário Procape, Recife 74970-240, PE, Brazil; dsobral@uol.com.br; 19 Hospital Universitário Antônio Pedro, UFF, Niterói 24033-900, RJ, Brazil; anobrega@id.uff.br; 20 Faculdade de Medicina de Ribeirão Preto, USP Ribeirão Preto, Ribeirão Prêto 14040-900, SP, Brazil; fernando.nobre@uol.com.br; 21 Imperial College London, London SW7 2AZ, UK; otavioberwanger@gmail.com

**Keywords:** left ventricular mass, electrocardiography, S-L voltage, Cornell product, treatment, hypertension, blood pressure

## Abstract

**Background:** The comparison of left ventricular mass (LVM) at different BP levels and the effects of antihypertensive drug treatment on LVM are unknown. **Objective:** To compare the LVM of individuals with prehypertension and Stage 1 hypertension and assess the effects of treatment on LVM at these stages of hypertension. **Methods:** We estimated LVM in the PREVER-Prevention trial using Sokolow–Lyon and Cornell voltage and voltage–duration products before and after randomization to 18 months of treatment with low doses of chlorthalidone and amiloride or placebo in adults with JNC 7 “prehypertension” (systolic BP [SBP] of 120–139 mm Hg and diastolic BP [DBP] of 80–89 mm Hg). Similarly, in the PREVER-Treatment trial, we assessed these indices before and after randomization to 18 months of treatment with the chlorthalidone/amiloride combination or losartan in adults with JNC 7 “stage 1” hypertension (140–159 mm Hg or DBP of 90–99 mm Hg). **Results:** At baseline, the participants in the stage I hypertension trial exhibited higher mean LVM indices than those in the prehypertension trial. In the PREVER-Prevention trial, those randomized to the chlorthalidone/amiloride combination experienced a significant reduction in Sokolow–Lyon LVM indices compared to placebo (*p* = 0.02). In the PREVER-Treatment trial, those randomized to the chlorthalidone/amiloride combination or losartan experienced a similar reduction in electrocardiographic LVM during the 18 months of treatment (*p* < 0.01). **Conclusions:** The institution of low-dose antihypertensive drug therapy in prehypertension and treatment of patients with stage 1 hypertension has the potential to interrupt the progress of hypertensive cardiomyopathy.

## 1. Introduction

Evidence of subclinical end-organ damage, such as an increase in left ventricular mass (LVM), optic fundus abnormalities, blood pressure (BP) variability, and high central BP, may refine cardiovascular disease (CVD) risk stratification in patients with hypertension [1]. Electrocardiographically detected left ventricular hypertrophy (LVH) was the first test employed to stratify cardiovascular risk in individuals with hypertension. In 274 men studied in the Framingham Heart Study, those without CVD who had evidence of LVH on electrocardiography at baseline experienced a substantial increase in mortality and CVD events during two years of follow-up [2]. When a serial change in ECG was considered, those with more pronounced LVH during follow-up had a higher risk of CVD. In contrast, their counterparts with a serial reduction in LVH experienced a significantly lower risk for CVD [3].

The prevention of LVH and reduction in pre-existing LVH have been explored in randomized controlled trials (RCTs) and observational analyses within RCTs. The HOPE trial demonstrated that ramipril reduced the incidence of electrocardiographic LVH in patients at high CVD risk independent of BP levels [4]. In addition, patients with ramipril-related reduction in LVH had a lower incidence of major CVD events [4]. In the LIFE study, antihypertensive therapy resulting in less severe electrocardiographic LVH was associated with reduced CVD morbidity and mortality, independent of BP lowering and treatment modality [5]. The TOMHS study demonstrated that chlorthalidone was superior to the lifestyle treatment of hypertension in reducing electrocardiographic LVM [6]. More recently, participants in the SPRINT who underwent intensive BP reduction (target systolic BP < 120 mmHg), compared to standard BP reduction (target systolic BP < 140 mmHg), exhibited significantly lower rates of LVH incidence in those without LVH at baseline and higher rates of LVH regression in those with LVH at baseline [7].

The natural history of LVM and its role as a CVD risk predictor and progression to LVH have received limited scrutiny. In 3661 Framingham Heart Study participants, both baseline echocardiographic LVM and LVH were associated with a significantly higher risk of sudden death during a mean follow-up of 10.3 years [8]. In 3042 Cardiovascular Health Study (CHS) participants, echocardiographic or ECG LVM at baseline was significantly associated with depressed LV ejection fraction during approximately 5 years of follow-up [9]. More recently, new-onset LVM over a 10-year period was associated with a statistically increased risk of CVD and all-cause mortality over 18.5 years of follow-up in 990 adults enrolled in the PAMELA cohort [10]. In the PREVER-Prevention [11] and PREVER-Treatment [12] trials, antihypertensive drug treatment reduced LVM.

Understanding the prognosis of LVM progression in patients with different stages of hypertension may help develop strategies to prevent the development of hypertensive cardiomyopathy. The primary goal of this analysis was to provide an integrated perspective on the effects of treatment on LVM at various stages of hypertension, examining the hypothesis that active treatment would lead to its reduction. In this report, we present the LVM trajectories and the effectiveness of interventions in reducing LVM in individuals with prehypertension and stage I hypertension.

## 2. Methods

Details of the PREVER-Prevention and PREVER-Treatment trials have been previously reported [11,12]. Both studies were randomized, controlled, double-blind clinical trials conducted at 21 academic medical centers in Brazil. The Ethics Committees of the participating centers approved the studies, which were registered on the clinicaltrials.gov website (NCT00970931 and NCT00971165).

The PREVER-Prevention trial [11] aimed to investigate if the use of an association of low doses of diuretics, chlorthalidone, and amiloride would reduce the incidence of hypertension in patients with prehypertension by the JNC 7 criteria (systolic BP between 120 and 139 mmHg and diastolic BP between 80 and 89 mmHg). Participants who were not being treated with antihypertensive medication but had received a three-month lifestyle change intervention that involved dietary advice and a recommendation to increase physical activity were randomized in a 1:1 ratio to receive daily single pill chlorthalidone 12.5 mg and amiloride 2.5 mg (n = 372) or placebo (n = 358). After randomization, follow-up visits were conducted after 3, 6, 9, 12, 15, and 18 months. The study’s primary objectives were to investigate the efficacy of treatment with the low-dose diuretic/amiloride combination for hypertension prevention, evaluate its safety, and examine the effects of the active treatment on end-organ damage. The reduction in the incidence of hypertension by close to 50% in 18 months was relevant, together with the finding that the therapy promoted a significant decrease in LVM estimated by ECG.

The main objective of the PREVER-Treatment trial was to compare the BP-lowering efficacy of the chlorthalidone and amiloride combination treatment with losartan during the initial management of patients with stage I hypertension [12]. Participants who had been managed with the previously mentioned three-month lifestyle counseling intervention but were not being treated with antihypertensive medication and had JNC 7 stage I hypertension, defined as an average systolic BP between 140 and 159 mmHg or diastolic BP between 90 and 99 mmHg, were randomized in a 1:1 ratio to receive daily losartan 50 mg (n = 322) or single pill chlorthalidone 12.5 mg combined with amiloride 2.5 mg (n = 333). If necessary, the doses of these treatments were doubled every 3 months, and additional drugs were added to control systolic/diastolic BP to <140/90 mmHg. After randomization, follow-up visits were conducted after 3, 6, 9, 12, 15, and 18 months. This study showed that the diuretic-based treatment of hypertension was more effective in lowering BP during 18 months of follow-up than the losartan-based treatment.

The effects of the treatments on LVM were defined as co-primary outcomes in both trials. In the PREVER-Prevention trial, the treatment with the combination of diuretics, but not placebo, reduced the LVM [11]. In the PREVER-Treatment trial, both treatments reduced the LVM [12].

In both trials, LVM was measured using Sokolow–Lyon and Cornell voltage and voltage–duration product ECG diagnostic criteria [13,14], which were calculated semi-automatically. Changes in these indices were calculated as the difference between the baseline and measurements taken after 18 months of treatment.

For this post hoc analysis, there was no a priori sample size calculation. Covariance analysis was conducted, adjusting for age and body mass index (BMI), which was calculated as weight (kg) divided by height (m) squared. Statistical analysis was performed using the SPSS software package version 21.0 (SPSS, Armonk, New York, NY, USA), and differences with a *p*-value < 0.05 were considered statistically significant.

## 3. Results

The main trial reports provided details on the number of individuals screened for the trials, reasons for exclusion, and the proportion of those who responded to lifestyle recommendations [11,12]. Table 1 displays a selected group of baseline demographic, clinical, and laboratory characteristics in the study groups, indicating similar measurements for the two treatment groups in each study. In addition to having a higher BP compared with their counterparts in the PREVER-Prevention trial, the participants in the PREVER-Treatment trial had a higher prevalence of diabetes mellitus. The initial dose of drugs in the PREVER-Treatment trial was more frequently doubled in the losartan arm than in the diuretic arm. In addition, BP-lowering drugs were used more frequently in the participants allocated to the losartan-based treatment than in the diuretic-based treatment.

Table 2 presents data on the electrocardiographic estimation of LVM for the four indices evaluated at the baseline in the PREVER participants with prehypertension and in those with stage I hypertension at the baseline. LVM was estimated using electrocardiographic indices in 1385 participants. In the PREVER-Prevention study, 60 participants in the chlorthalidone and amiloride group and 68 in the placebo group discontinued their participation during follow-up, mostly due to the development of hypertension, the main trial outcome [9]. In the PREVER-Treatment study, 23 participants in the chlorthalidone and amiloride group and 23 in the losartan group discontinued their participation, primarily due to the withdrawal of consent (n = 16) or loss to follow-up (n = 7). Compared to the participants with stage 1 hypertension, those with prehypertension had lower mean LVM values for all of the indices measured, with statistically significant differences in three of the four measurements (Sokolow–Lyon voltage–duration product, Cornell voltage, and Cornell voltage–duration product) and a trend that was close to being statistically significant for the fourth measurement (Sokolov–Lyon voltage).

Table 3 details the mean estimates of LVM according to the four electrocardiographic indices analyzed in the PREVER-Prevention study comparing the baseline values with those after 18 months of intervention. The indices are separated into intervention and placebo groups. Sokolow–Lyon indices identified a statistically significant reduction in LVM for the intervention group without a corresponding change in the placebo group. After 18 months, there was a statistically significant difference between the intervention and control groups (*p* = 0.02 for both Sokolow–Lyon indices).

Although LVM estimates for the Cornell voltage and Cornell voltage–duration product indices demonstrated a trend for reduction during follow-up, it was not statistically significant when the intervention and control groups were compared after 18 months of treatment (*p* = 0.17 and *p* = 0.07, respectively).

Table 4 presents an analysis of LVM changes based on electrocardiographic indices in the PREVER-Treatment study participants. The indices are presented separately for each group and its respective intervention: losartan or chlorthalidone combined with amiloride. The electrocardiographic indices of LVM decreased during the 18-month follow-up in both groups compared to the baseline without a statistically significant difference between the two study treatment arms.

**Figure 1:**
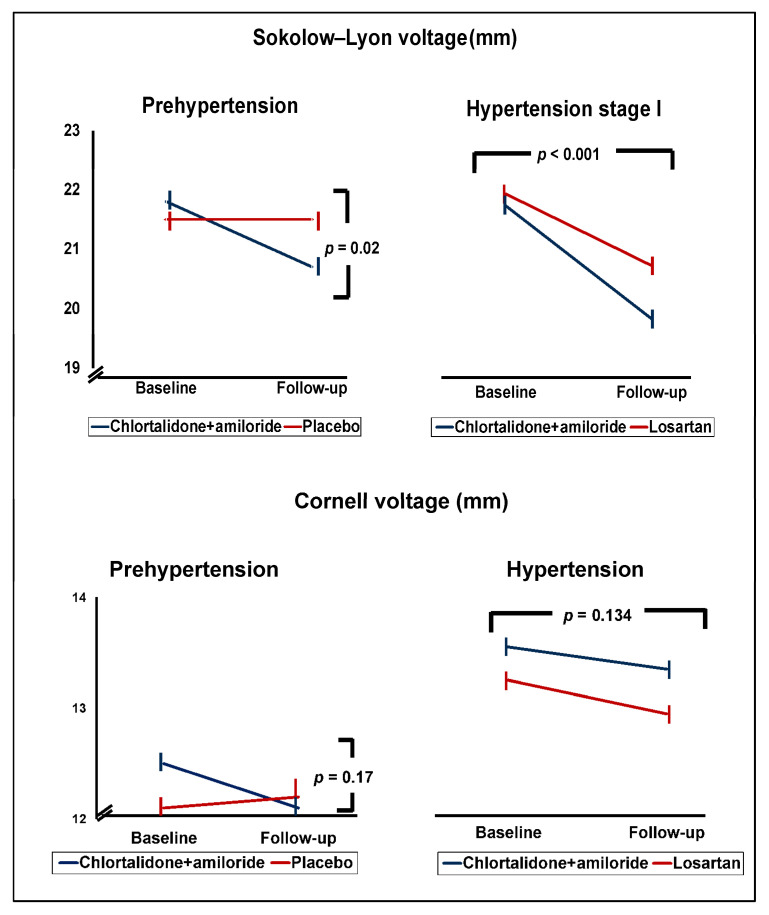
LVM trajectories and treatment effects in patients with prehypertension and hypertension estimated by the Sokolow–Lyon and Cornell voltage criteria.

Figure 1 displays the LVM changes estimated by the Sokolow–Lyon and Cornell voltage indices after 18 months of intervention. In the participants with prehypertension, there was a reduction in LVM using both diagnostic indices in the intervention group, with no corresponding decrease in the placebo group. Consequently, the estimated LVM using both indices was lower in the participants in the intervention group than in the placebo group at the end of the follow-up period, with statistical significance for the difference in the Sokolow–Lyon voltage index (*p* = 0.02). In the patients with stage I hypertension, both the Sokolow–Lyon voltage index and the Cornell voltage index LVM measurements were reduced in both treatment arms—the losartan group and the chlorthalidone plus amiloride group—with statistical significance for the difference between the baseline and the 18-month follow-up in the Sokolow–Lyon voltage index (*p* < 0.001).

## 4. Discussion

This analysis provides original insights at two different time points for LVM in adults with two categories of BP and the influence of treatment on the measurements. First, the static comparison of the LVM values showed that the individuals with prehypertension had less LVM than those with JNC 7 stage I hypertension. Second, the dynamic comparison of the LVM values demonstrated that treatment with a low-dose combination of chlorthalidone and amiloride in adults with prehypertension and full doses of the chlorthalidone and amiloride combination or losartan in adults with stage I hypertension reduced LVM. These findings showed that drug treatment could effectively reverse the pathological adaptive ventricular response to higher levels of BP, preventing the long-term consequences of hypertensive cardiomyopathy [15].

Our findings were obtained in two studies conducted simultaneously under similar conditions and employing similar methods. Both studies were randomized, double-blind clinical trials with the same duration, allocation concealment, intention-to-treat analysis, and electrocardiographic index measurements performed using identical methods. Additionally, both studies included many participants from various regions of Brazil.

No study has integrated the course of LVM by stages of hypertension and demonstrated a lower LVM in individuals with JNC 7 prehypertension compared to those with stage 1 hypertension. In subsamples of the PREVER-Prevention and -Treatment trials, the patients with stage I hypertension exhibited a lower global longitudinal strain (GLS) than individuals with prehypertension [16]. This difference in GLS has been linked to a higher risk of cardiovascular disease [17]. Lower GLS was also reported in individuals with masked hypertension when compared to those who are normotensive. [18].

In adults with hypertension, previous studies have reported a reduction in the incidence of LVH with antihypertensive drug treatment [4,6]. This decrease in LVH has been associated with a lower incidence of CVD events [4,5]. In a subsample of participants from the PREVER-Treatment trial who had undergone echocardiographic exams before and after treatment with drug strategies, there was a favorable remodeling in the LVM index and relative wall thickness, increasing the proportion of participants with normal LV geometry [19]. Our findings extend the knowledge of ECG observations in adults with prehypertension and hypertension who had evidence of LVM but no LVH, strengthening the hypothesis that the level of blood pressure influences the prevalence of LVM and earlier pharmacological treatment in individuals with prehypertension and stage I hypertension could anticipate and prevent an important indicator of CVD risk.

Prehypertension has been associated with increased echocardiographic left ventricular remodeling and impaired diastolic function [20]. The reduction in LVM with treatment in the PREVER-Prevention trial suggests that it may favorably influence the progression of cardiac consequences in individuals with BP within the 120 and 139 mmHg range. The SPRINT experience with intensive treatment to an SBP target of less than 120 mmHg [21] underscores the safety of intensive pharmacological intervention to the level of blood pressure in the PREVER-Prevention trial [11].

An integrated view of our findings is that BP-lowering therapies can effectively prevent the increase in LVM caused by high BP. This effect will likely reduce the incidence of consequences related to hypertensive cardiomyopathy, such as heart failure with preserved ejection fraction and atrial fibrillation.

Other ECG abnormalities besides LVH can be explored to identify individuals at higher risk of developing CVD, as the occurrence of premature ventricular beats has been associated with high blood pressure [22,23].

In conclusion, increases in left ventricular mass are a continuous phenomenon associated with increasing BP and can be reversed by antihypertensive drug treatment at different BP levels. Early treatment of high BP has the potential to prevent long-term consequences, particularly those secondary to hypertensive cardiomyopathy.

## Figures and Tables

**Table 1:** Baseline characteristics of the PREVER-Prevention and PREVER-Treatment trial participants.

	PREVER-Prevention		PREVER-Treatment	
	Intervention (N = 372)	Placebo (N = 358)	Chlorthalidone + Amiloride (N = 333)	Losartan (N = 322)
Male	186 (50.0)	179 (50.1)	167 (50.2)	167 (51.9)
Age (years)	50 ± 10	50 ± 11	53.9 ± 8.4	54.7 ± 7.9
White ^1^	195 (52)	206 (58)	205 (61.6)	198 (61.5)
Education (years)	11 ± 4	11 ± 4	10.7 ± 4.6	10.5 ± 4.2
Body mass index (kg/m^2^)	29 ± 5	29 ± 5	29.1 ± 5	28.8 ± 4.7
Systolic BP (mmHg)	128 ± 7	128 ± 7	142.6 ± 7.1	142.1 ± 6.5
Diastolic BP (mmHg)	81 ± 6	80 ± 6	89.7 ± 6.3	89.4 ± 6.1
Cholesterol (mg/dl)	193 ± 37	193 ± 41	196.8 ± 40.5	193.2 ± 39.1
Creatinine (mg/dl)	0.8 ± 0.2	0.8 ± 0.2	0.8 ± 0.18	0.8 ± 0.19
Diabetes mellitus ^2^ (%)	30 (8)	29 (8)	47 (14.2)	50 (15.6)
Current smokers	28 (8)	37 (10)	27 (8.1)	21 (6.5)
Current alcoholic consumption	227 (61)	206 (58)	223 (67.0)	197 (61.2)

Data are shown as n (%) or mean ± SD. ^1^ self-reported and categorized as white or nonwhite. ^2^ previous physician’s diagnosis, use of antidiabetics, abnormal fasting glucose, or glycosylated hemoglobin at baseline.

**Table 2:** Electrocardiographic indices at the baseline of participants in the PREVER-Prevention and PREVER-Treatment studies, controlling for age and BMI.

ECG Index	PREVER-Prevention	PREVER-Treatment	Delta (95%CI)	*p* Value
SLV (mm ^a^)	21.6 ± 0.3	22.3 ± 0.3	−0.8 (−1.6 to 0.03)	0.058
SLVD ^a^ (µVms)	225.0 ± 3.6	237.2 ± 4	−12.2 (−23.0 to −1.4)	0.026
CV (mm ^a^)	12.4	13.5 ± 0.2	−1.1 (−1.7 to −0.5)	<0.001
CDV ^a^ (µVms)	128.8 ± 2.5	142.0 ± 2.7	−13.2 (−20.6 to −5.8)	<0.001

^a^ 1 mm = 0.1 mV. SLV: Sokolow–Lyon voltage; SLVD: Sokolow–Lyon voltage–duration product; CV: Cornell voltage; CVD: Cornell voltage–duration product.

**Table 3:** Variation in ECG indices of left ventricular hypertrophy by treatment group (PREVER-Prevention).

ECG Index	Group (n)	Baseline	Visit 18	Delta (95%CI)	*p* *
SLV (mm ^a^)	Chlorthalidone/amiloride (251)	21.8 ± 7.5	20.7 ± 7.1	1.1 (0.4 to 1.7)	0.02
	Placebo (257)	21.5 ± 7.4	21.5 ± 7.2	0.03 (−0.59 to 0.64)	
SLVD ^a^ (µVms)	Chlorthalidone/amiloride (246)	229.0 ± 102.7	213.3 ± 83.6	15.4 (5.7 to 25.2)	0.02
	Placebo (253)	223.0 ± 98.6	224.4 ± 94.4	−1.4 (−11.1 to 8.2)	
CV (mm ^a^)	Chlorthalidone/amiloride (252)	12.5 ± 5.3	12.1 ± 5.0	0.04 (−0.01 to 0.09)	0.17
	Placebo (259)	12.1 ± 5.0	12.2 ± 5.0	−0.01 (−0.05 to 0.04)	
CDV ^a^ (µVms)	Chlorthalidone/amiloride (247)	129.5 ± 65.5	122.8 ± 56.3	6.9 (−0.1 to 13.9)	0.07
	Placebo (254)	125.3 ± 63.7	127.7 ± 69.0	−2.4 (−9.3 to 4.6)	

Patients with valid ECGs at the baseline evaluation and at visit 18; there are fewer patients in the voltage duration indices because of the imprecision in the measurement of QRS duration. *p*, for interaction time: treatment. * analysis using a *t*-test for independent samples for the between-group difference. ^a^ 1 mm = 0.1 mV.

**Table 4:** Variation in ECG indices of left ventricular hypertrophy by treatment group (PREVER-Treatment).

ECG Index	Group (n)	Baseline	Visit 18	Delta (95%CI)	*p* *
SLV (mm ^a^)	Chlorthalidone/amiloride (250)	21.8 ± 6.5	19.9 ± 6.1	1.8 (1.3 to 2.3)	0.153
	Losartan (234)	22.0 ± 8.2	20.8 ± 7.3	1.3 (0.7 to 1.8)	
SLVD ^a^ (µVms)	Chlorthalidone/amiloride (235)	230.7 ± 89.6	210.2 ± 82.2	20.4 (12.3 to 28.6)	0.821
	Losartan (223)	236.8 ± 104.7	215.0 ± 95.1	21.8 (13.4 to 30.1)	
CV (mm ^a^)	Chlorthalidone/amiloride (250)	13.5 ± 5.1	13.3 ± 5.0	0.2 (−0.3 to 0.7)	0.743
	Losartan (234)	13.2 ± 5.2	12.9 ± 5.5	0.3 (−0.2 to 0.8)	
CDV ^a^ (µVms)	Chlorthalidone/amiloride (235)	141.5 ± 61.4	138.2 ± 57.2	3.3 (−3.2 to 9.9)	0.163
	Losartan (223)	141.2 ± 66.0	131.2 ± 61.5	10.0 (3.3 to 16.7)	

Patients with valid ECGs at the baseline evaluation and at visit 18; there are fewer patients in the voltage duration indices because of the imprecision in the measurement of QRS duration. *p*, for interaction time: treatment. * analysis using a *t*-test for independent samples for the between-group difference. ^a^ 1 mm = 0.1 mV.

## Data Availability

The data analyzed in this study were obtained from previously published studies and are, therefore, not available as a single dataset. Individual study data can be accessed through the cited publications.
